# Depolymerisation of the *Klebsiella pneumoniae* Capsular Polysaccharide K21 by Klebsiella Phage K5

**DOI:** 10.3390/ijms242417288

**Published:** 2023-12-09

**Authors:** Anna A. Lukianova, Mikhail M. Shneider, Peter V. Evseev, Mikhail V. Egorov, Anastasiya A. Kasimova, Anna M. Shpirt, Alexander S. Shashkov, Yuriy A. Knirel, Elena S. Kostryukova, Konstantin A. Miroshnikov

**Affiliations:** 1 Shemyakin-Ovchinnikov Institute of Bioorganic Chemistry, Russian Academy of Sciences, Miklukho-Maklaya Str. 16/10, 117997 Moscow, Russia; petevseev@gmail.com (P.V.E.); mikhail-egorov-1998@mail.ru (M.V.E.); kmi@bk.ru (K.A.M.); 2 Zelinsky Institute of Organic Chemistry, Russian Academy of Sciences, Leninsky prosp. 47, 119991 Moscow, Russia; nastia-kasimova979797@mail.ru (A.A.K.); asyashpirt@gmail.com (A.M.S.); alexander.shashkov@mail.ru (A.S.S.); yknirel@gmail.com (Y.A.K.); 3 Lopukhin Federal Research and Clinical Center of Physical-Chemical Medicine, Federal Medical Biological Agency, Malaya Pirogovskaya Str. 1, 119435 Moscow, Russia; el-es@yandex.ru

**Keywords:** *Klebsiella pneumoniae*, bacteriophage, phage depolymerases

## Abstract

*Klebsiella pneumoniae* is a pathogen associated with various infection types, which often exhibits multiple antibiotic resistance. Phages, or bacterial viruses, have an ability to specifically target and destroy *K. pneumoniae*, offering a potential means of combatting multidrug-resistant infections. Phage enzymes are another promising therapeutic agent that can break down bacterial capsular polysaccharide, which shields *K. pneumoniae* from the immune response and external factors. In this study, *Klebsiella* phage K5 was isolated; this phage is active against *Klebsiella pneumoniae* with the capsular type K21. It was demonstrated that the phage can effectively lyse the host culture. The adsorption apparatus of the phage has revealed two receptor-binding proteins (RBPs) with predicted polysaccharide depolymerising activity. A recombinant form of both RBPs was obtained and experiments showed that one of them depolymerised the capsular polysaccharide K21. The structure of this polysaccharide and its degradation fragments were analysed. The second receptor-binding protein showed no activity on capsular polysaccharide of any of the 31 capsule types tested, so the substrate for this enzyme remains to be determined in the future. *Klebsiella* phage K5 may be considered a useful agent against *Klebsiella* infections.

## 1. Introduction

*Klebsiella pneumoniae* (*Kpn*) is a Gram-negative bacterium that is an important pathogen causing human infections. *Kpn* is capable of colonising various organs, including lungs, the urinary tract, blood, wounds and the liver [1]. In particular, *Kpn* spreads as a nosocomial infection in hospitals, contaminating catheters, medical gloves and the clothing of medical employees [2].

Moreover, *K. pneumoniae* is also often characterised by multiple drug resistance (MDR), caused by various genetic mechanisms. Numerous plasmids have been identified for *Kpn* that possess the determinants of resistance to carbapenems, cephalosporins, fluoroquinolones, aminoglycosides and other antibiotic groups [3,4]. MDR may develop due to a number of different mechanisms, such as the production of specific antibiotic-degrading enzymes (e.g., β-lactamases), decreased cell permeability, active transport of the antibiotic out of the cell via efflux pumps, or through target modification [5].

Naturally, *Kpn* cells are surrounded by a capsule of extracellular polysaccharide (capsular polysaccharide, CPS), which enables them to both hide from the host’s immune system and resist external factors, including antimicrobial compounds [6]. CPS enhances the ability of *Kpn* to form biofilms. Thus, hypermucoid strains are especially dangerous, since they are much more virulent than isolates with fewer CPS [7]. Historically, 79 types of capsular polysaccharide types have been identified serologically in *Kpn*; at the moment, 186 genetic loci encoding capsular polysaccharide are recognised [8,9].

These features make *Kpn* extremely adaptive and invasive, causing serious infections that may be difficult to treat.

When the treatment of multidrug-resistant infections is required, the use of bacteriophages, or phage therapy, can be an effective addition to traditional antibiotics. Bacteriophages are viruses that specifically attack and destroy bacteria. This approach is also suitable for the treatment of infections caused by *Kpn*. For example, phage therapy has shown positive results in the treatment of liver abscesses [10], pneumonia [11,12] and systemic bacteremia [13] in a mouse model. In addition, successful cases of using phage therapy in combination with antibiotics and on patients with *Klebsiella* infections have been reported [14,15].

Some bacteriophages have an enzyme apparatus enabling them to destroy the *Kpn* capsule. Depolymerising the exopolysaccharide facilitates the bacteriophage’s access to the cell. Specific polysaccharide depolymerases are of great scientific interest from both a fundamental and applied perspective. In particular, the study of phage depolymerases provides insights into the details of phage–cell interaction. In addition, these enzymes can be considered as a therapeutic agent in anti-*Klebsiella* therapy per se. Thus, the use of phage depolymerases has been shown to have caused an effective reduction in biofilm formation in vitro [16,17], a protective effect against *Galleria mellonella* larvae in a model infection [18] and promising therapeutic effects in mice [19,20]. This suggests that the search for new bacteriophages active against *Kpn*, and the study of their biological properties, adsorption apparatus and, in particular, phage depolymerases, is an important research task.

In this work, the bacteriophage K5, which is active against *Kpn* strain with K21 CPS type, was isolated; then, an in silico study of its taxonomic position was conducted and its adsorption apparatus was modelled. Recombinant receptor-binding proteins (RBPs) of this phage with predicted polysaccharide-degrading activity were obtained. In addition, the chemical mechanism of cleavage of K21 CPS by phage depolymerase was investigated.

## 2. Results

### 2.1. General Biological Properties

*Klebsiella* phage K5 was isolated in 2015, from wastewater collected in the Moscow region (Russia), using the *K. pneumoniae* strain 5 with the capsule type K21.

**Figure 1:**
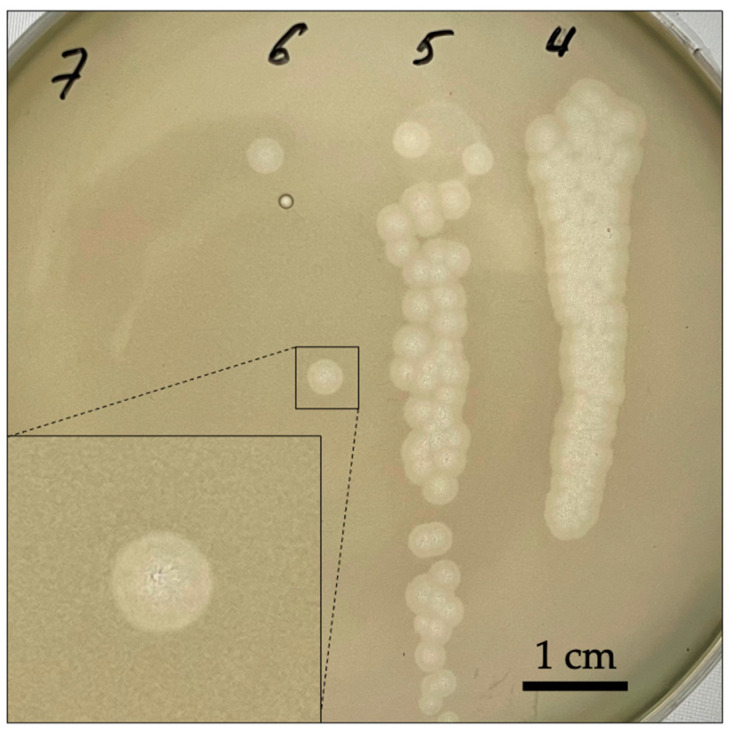
Plaques formed by bacteriophage K5 on 0.75% agar. The numbers on the Petri dish indicate the number of the tenfold dilution applied to the dish.

The bacteriophage formed large plaques 1.5–2 mm in diameter, with a wide halo on 0.75% top agar (Figure 1). The presence of a translucent halo around the plaque is frequently indicative of the presence of phage capsular polysaccharide depolymerase.

**Figure 2:**
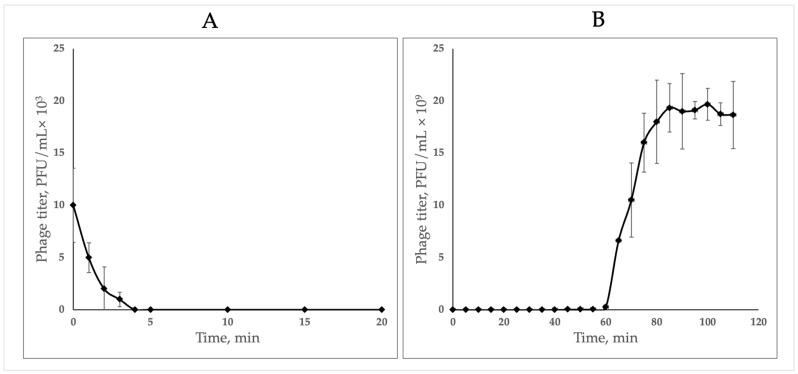
(**A**) Adsorption of *Klebsiella* phage K5 to isolation host, MOI = 0.001; (**B**) one-step growth curve on isolation host, MOI = 0.01.

The bacteriophage quickly adsorbed to the host cells and after 4 min of incubation (Figure 2A) no unbound phage particles were observed in the medium. Adsorption was followed by a latent period of 25 min, when a gradual release of phage particles started, followed by a rapid increase in free phage particles in the medium, reaching a plateau with a final titer of 1.9 × 10^10^ PFU/mL (burst size 160 ± 10 PFU/cell) (Figure 2B).

The host range of *Klebsiella* phage K5 was tested on a set of clinical isolates with different K-types (Table 1). The characterised abundant clinical isolates included strains with K1, K2, K12, K14, K16, K17, K20, K21, K23, K24, K39, K62, K64, K107, K108, K112 and K161 capsule types, and some that were not characterised.

Among the tested strains, *Klebsiella* phage K5 lysed only the isolation host with the K21 capsular type.

### 2.2. General Characterisation of Genome

*Klebsiella* phage K5 (GenBank accessions #KR149291 for initial submission in 2015 and #NC_028800 for refined re-annotated version in 2023) has a linear double-stranded DNA genome. The K5 genome size is 41,698 base pairs (bp) and the average GC content of the genome is 52.5%, which is slightly less than the typical GC content of *Kpn* of 57%. Forty-six open-reading frames (ORFs) and no tRNA genes were found in the genome. Direct terminal repeats, 392 bp long, flank the genome. Putative functions were suggested for 34 ORFs; 12 ORFs were annotated as encoding hypothetical proteins.

**Figure 3:**
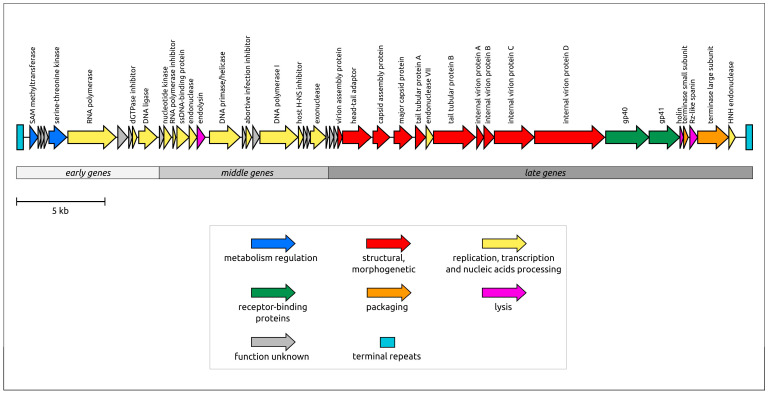
Genetic maps of *Klebsiella* phage K5. Arrows indicate the direction of transcription. The scalebar indicates the length of the nucleotide sequence. Gene functions and modules are shown in labels and legends.

The general architecture of the genome (Figure 3) is similar to that of T7-like phages belonging to the *Studiervirinae* subfamily of the *Autographiviridae* family. Like other *Autographiviridae* genomes, the K5 genome contains genes for RNA polymerase, involved in the transcription of early genes, and phage DNA polymerase, responsible for replication [21]. The block of structural and morphogenetic genes encodes the HK97 major capsid protein (MCP) and other virion proteins, including two receptor-binding proteins, gp40 (gene product 40) and gp41. Phage lysis machinery comprises endolysin, holin and spanin [22].

### 2.3. Taxonomy and Phylogeny

#### 2.3.1. Related Phages

**Figure 4:**
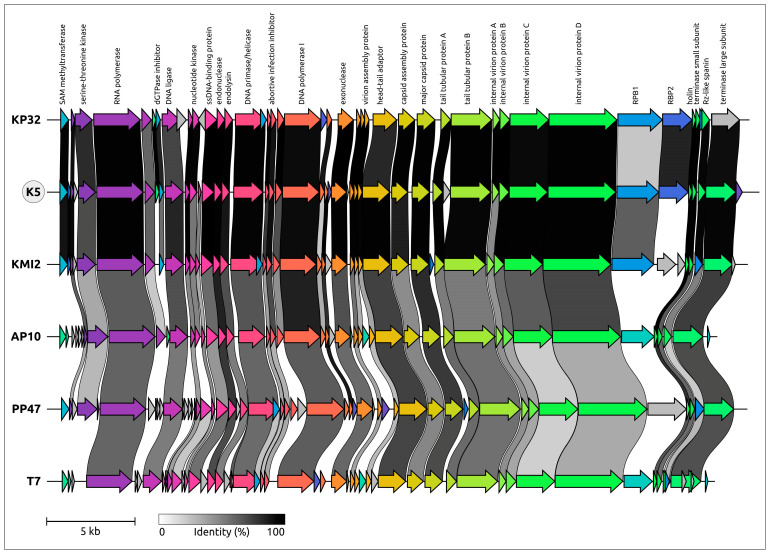
Comparative genome alignment of *Klebsiella* phage K5 (circled) and phages *Klebsiella* phage KP32 (KP32), *Klebsiella* phage KMI2 (KMI2), *Yersinia* phage vB_YenP_AP10 (AP10), *Pectobacterium* phage PP47 (PP47) and *Escherichia* phage T7 (T7). The percentage of amino acid identity is represented by grey-scaled links between genomes, as explained in the legend. Homologous proteins are assigned a unique colour. Gene functions are shown in labels.

A search for related phages was conducted using gene sequences encoded in the K5 genome. Revealed closest relatives were among the phages assigned to the *Przondovirus* genus (*Studiervirinae* subfamily, *Autographiviridae* family) infecting *Klebsiella*. Comparative genome alignment indicated a high level of gene synteny between phage K5 and *Przondovirus* phages (KP32-like phages) (Figure 4). Except for a few cases, the genes of K5 and *Przondovirus* phages KP32 and KMI2 showed pronounced homology. Compared with most conserved proteins, this homology is lower, or missing, for receptor-binding proteins (gp40 and gp41 in K5). Phages infecting other Enterobacteria and assigned to other *Studiervirinae* genera (*Yersinia* phage vB_YenP_AP10 [23], *Pectobacterium* phage PP47 [24], *Escherichia* phage T7) showed less gene identity with K5. However, the genome architecture of all analysed phages was essentially the same.

#### 2.3.2. VIRIDIC Intergenomic Similarity

**Figure 5:**
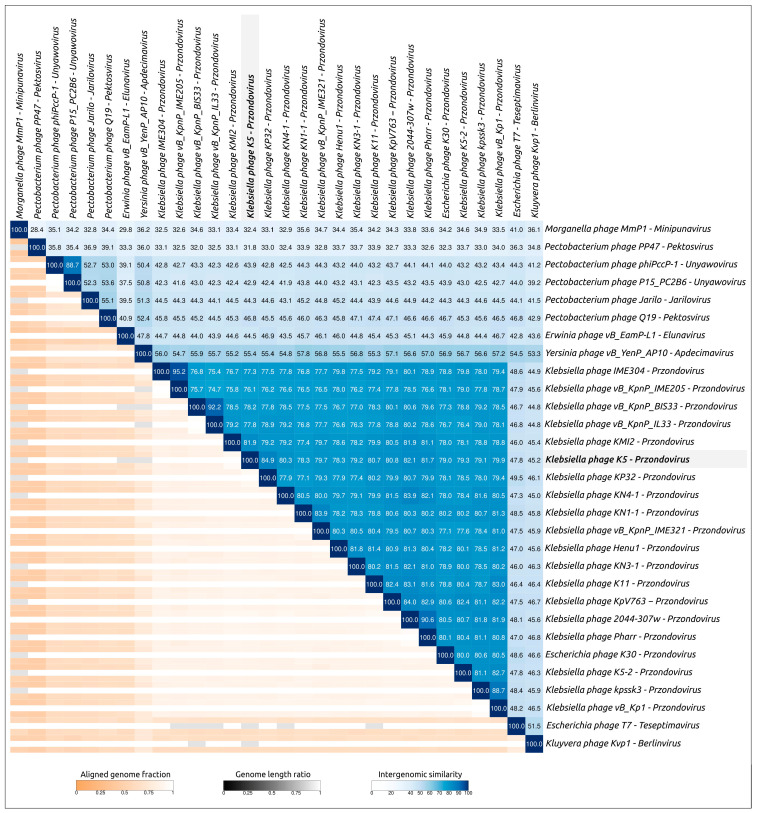
VIRIDIC-generated heatmap based on the genomic nucleotide-based intergenomic similarity of *Klebsiella* phage K5 and related phages. The colour coding in the upper-right part of the map indicates the clustering of the phage genomes based on intergenomic similarity. Numbers represent similarity values for each genome pair, rounded to the first decimal. The aligned genome fraction and genome length ratio are shown in the lower-left of the map, using a colour gradient that is explained in the legends.

To clarify the closest relatives of phage K5, calculations of nucleotide-based intergenomic similarity were performed using the comparative phage analysis tool VIRIDIC. VIRIDIC employs a classification technique acknowledged and recommended by the International Committee on Taxonomy of Viruses (ICTV) [25]. Genomic sequences of phages assigned to the *Przondovirus* genus and representatives of several other genera belonging to the *Studiervirinae* subfamily were used for the calculations. The VIRIDIC clustered heatmap (Figure 5) indicated a high level of intergenomic similarity between phage K5 and *Przondovirus* phages, of 75–85%, which is above the 70% genus boundary. Nevertheless, the genomic similarity between phage K5 and its closest relatives is below 95%, and thus it can be assigned as a separate taxonomic species.

#### 2.3.3. Phylogenetic Analysis

**Figure 6:**
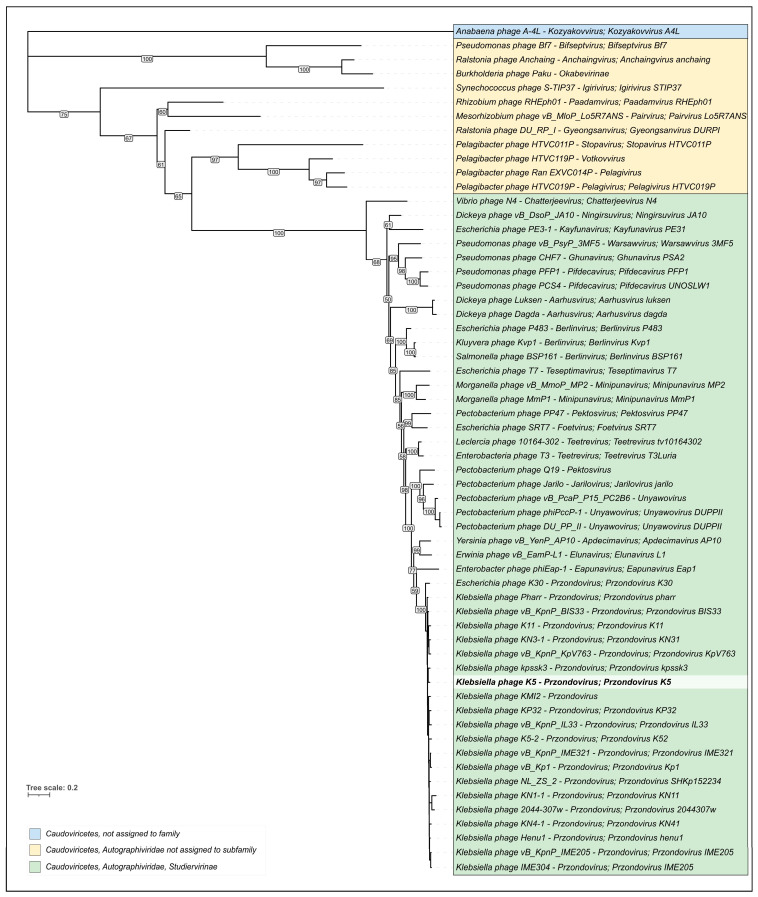
Phylogenetic trees based on nucleotide sequences of major capsid protein. Phage taxonomy is shown to the right of the phage name. Bootstrap values are shown near their branches. Branches with bootstrap support lower than 50% were deleted. The scalebar shows 0.2 estimated substitutions per site, and the tree was rooted to *Anabaena* phage A-4L.

Phylogenetic analysis was conducted using MCP nucleotide sequences of representatives of *Autographiviridae* phages. The phylogenetic tree groups phage K5 and phages belonging to the *Przondovirus* genus into one clade (Figure 6). The monophyleticity of the branch comprising phage K5 and *Przondovirus* phages, together with the results of the VIRIDIC analysis, suggest the assignment of *Klebsiella* phage K5 to the genus of *Przondovirus*. According to the results of intergenomic comparison and phylogenetic analysis, phage K5 is quite close to phage KP32, which was previously considered as the type species of the *Przondovirus* genus (https://ictv.global/taxonomy/taxondetails?taxnode_id=202200555, accessed on 24 September 2023).

### 2.4. In Silico Analysis of K5 RBPs

**Figure 7:**
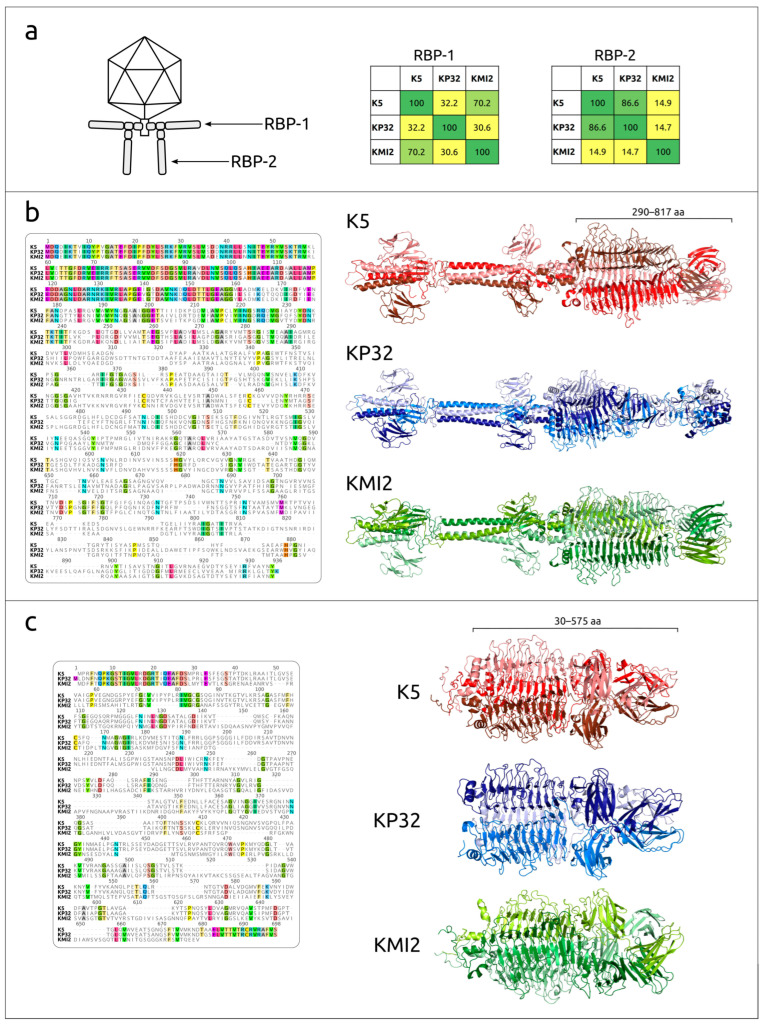
(**a**) Schematic view of KP32-like phage virions according to [28] and pairwise identity matrix of RBPs of *Klebsiella* phages K5, KP32 and KMI. (**b**) Sequence alignment and AlphaFold [29,30] models of RPB-1 of *Klebsiella* phages K5, KP32 and KMI. (**c**) Sequence alignment and AlphaFold models of RPB-2 of *Klebsiella* phages K5 and KMI and experimental structure of RBP-2 of *Klebsiella* phages KP32 (PDB code 6TKU).

Kp32-like viruses (*Przondovirus*) are characterised by the presence of two trimeric receptor-binding proteins (RBPs) that are active against specific types of capsular polysaccharide [26]. The genome of *Klebsiella* phage K5 encodes two proteins with predicted polysaccharide-degrading activity: gp40 (RBP-1) and gp41 (RBP-2) (Figure 7a). Gp40 is an 817 amino acid (aa) protein. It has an N-terminal anchor for attachment to the phage tail, an adjacent domain where a second RBP is attached and a polysaccharide-degrading domain. The first two domains occupy about a third of the sequence length (about 1–290 aa) (Figure 7b). A remote protein homology search using HHpred and comparisons of experimental and modelled structures of different RPB-2 indicated that phage K5 RBP-2 (gp41) has a short N-terminal binding domain involved in the attachment of RBP-2 to RBP-1 and a larger putative polysaccharide-degrading domain (Figure 7c).

Sequence comparisons revealed that the RPB-1 of phage K5 and KP32 showed no noticeable homology (32.2% pairwise identity, Figure 7a). Phage K5 RBP-1 is more similar to an RBP-1 of another *Przondovirus* phage, KMI2 (70.2% pairwise identity). At the same time, the RPB-2 of phage KMI2 is dissimilar, by its amino acid sequence, to the K5 RBP-2 (14.9% pairwise identity), but the KP32 RBP-2 (PDB code 6TKU) is very similar to RBP-2 of phage K5 (86.6% pairwise identity). It has been shown that the RBP-2 of phage KP32 is active against capsular type K21 [27,28].

### 2.5. Cloning and Expression of Putative Capsule Depolymerases

Both genes were cloned to pTSL vector and expressed in *E. coli* B834(DE3), producing proteins of the expected size for further study. The proteins were purified using a combination of N-chelating and anion-exchange chromatography.

**Figure 8:**
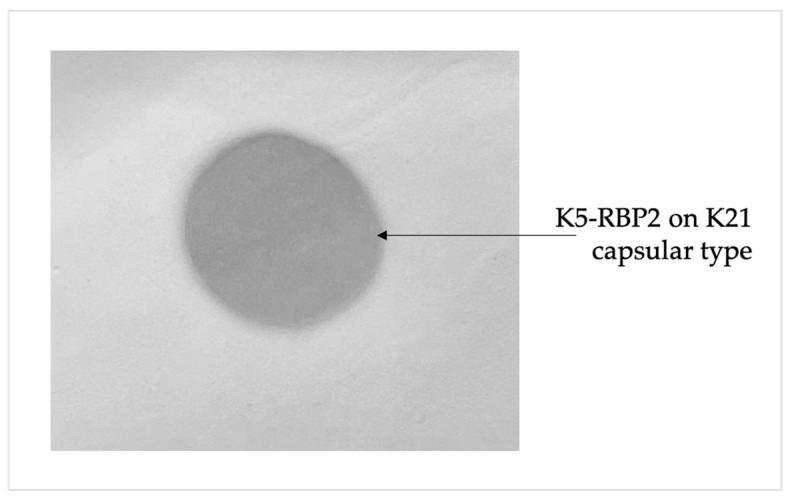
The spot assay with RBP2 depolymerase on *K. pneumoniae* strain capsular types of K21.

RBP2 exhibited polysaccharide degrading activity only on capsular polysaccharide type 21, while RBP1 showed no activity on any capsular type tested. Applying a purified protein preparation to a bacterial lawn led to the appearance of a translucent spot on the site of application (Figure 8).

### 2.6. Structure of the Capsular Polysaccharide and Its Cleavage by RBP2

The CPS was isolated from cells of *Kpn* strain 5 by phenol-water extraction [31]. Sugar analysis of the CPS by GLC of the acetylated alditols revealed mannose (Man) and galactose (Gal) in the ratio 1:1.3 (GLC detector response). The d configuration of Gal and Man was established using the ^13^C NMR data of the CPS using known regularities in the glycosylation effect [32].

The ^13^C NMR spectrum of the polysaccharide demonstrated a regular structure. It showed signals for five anomeric carbons at δ 95.9–104.2, pyruvic acid acetal at δ 101.5 (C-2) and δ 26.4 (CH_3_) attached to α-d-galactose; other sugar-ring carbons at δ 62.3–80.4, and two CO groups at δ 175.1 (C-1 of pyruvate) and 176.3 (C-6 of GlcA) (see Table 2). The ^1^H NMR spectrum contained, *inter alia*, signals for five anomeric protons at δ 4.85–5.50 and CH_3_ of pyruvate at δ 1.46 [33].

The α configuration of the glycosidic linkages of four sugar residue, including GlcA, two Man and Gal (units A, B, C and E, respectively), was established by *J*_1,2_ coupling constant values of 3.0–4.0 Hz. This conclusion was confirmed by the absence of H-1,H-3 and H-1,H-5 correlations in the 2D ROESY spectrum of the polysaccharide. The β-configuration for unit D followed from the H-1/H-3 and H-1H-5 correlations in the same spectrum [34] and *J*_1,2_ coupling constant values of 7.9 Hz.

Relatively low field positions of the signals for C-3 of units A at δ 80.5 C-4 of unit A at δ 71.3, C-3 of unit B at δ 79.6 and C-3 of unit D at δ 77.8, C-2 of unit C at δ 80.3, respectively, indicated the glycosylation pattern in the repeating unit [35].

The ROESY spectrum of the polysaccharide showed inter-residue cross-peaks between the anomeric protons and protons at the linkage carbons, which, taking into account the positions of glycosylation of the monosaccharides (see above), could be interpreted as follows: α-GlcA (A) H-1/α-Man (B) H-3 at δ 5.25/3.91; α-Man (B) H-1/α-Man (C) H-2 at δ 5.02/4.01, α-Man (C) H-1/β-Gal (D) H-3 at δ 5.23/3.76, β-Gal (D) H-1/α-GlcA (A) H-3/ at δ 4.85/4.31 and α-Gal (E) H-1/α-GlcA (A) H-4 at δ 3.92. These data confirmed the glycosylation pattern and established the monosaccharide sequence in the repeating unit.

**Figure 9:**
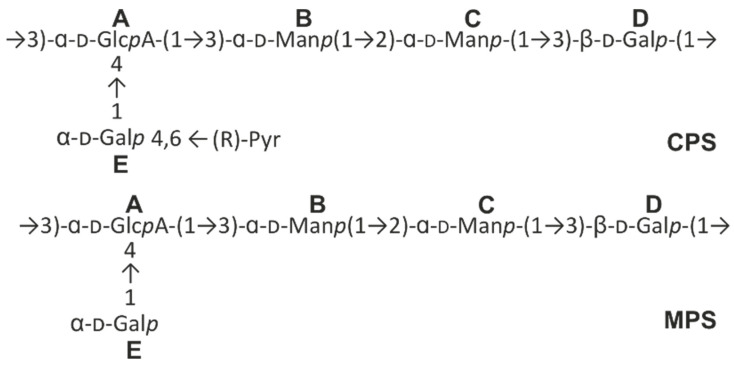
Structures of the capsular polysaccharide of *Kpn* 5 (CPS) and a modified polysaccharide without pyruvate group (MPS).

These data established the following structure of the capsular polysaccharide of *K. pneumoniae* K5 (Figure 9):

The structure of *Klebsiella* CPS type K21 has previously been presented in the literature [36], but this paper included insufficient details of ^1^H and ^13^C NMR spectra.

**Figure 10:**
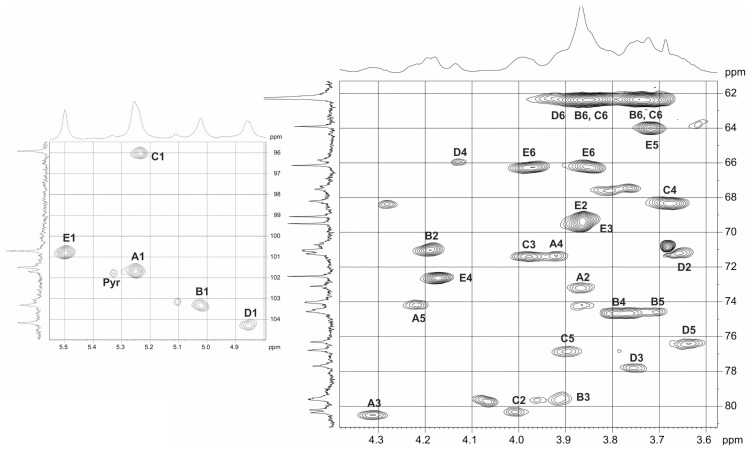
Parts of the ^1^H,^13^C HSQC spectrum of the *Kpn* K21 CPS. Arabic numerals refer to carbons of sugar residues, as designated in Table 2.

The intact polysaccharide of *Kpn* 5 (CPS type K21) was treated with 2% AcOH (7 h) for cleavage of the pyruvate group, followed by GPC of the carbohydrate portion on the TSK HW-40 column. The structure of the resultant polysaccharide (MPS) was established by the ^1^H and ^13^C NMR spectra; the chemical shifts are given in Table 2 (Figure 10).

**Figure 11:**
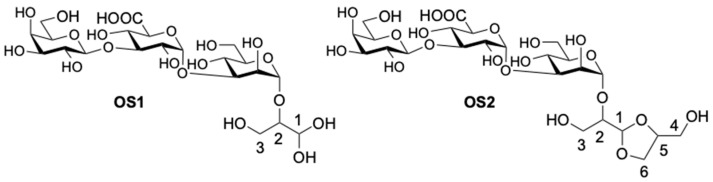
The chemical structures of oligosaccharides derived after Smith degradation of *Kpn* K21.

Therefore, the modified polysaccharide of the *Kpn* K21 had the structure shown in Figure 9, which was confirmed independently by Smith degradation. The resultant oligosaccharides (**OS1** and **OS2**) were isolated by GPC; their structures, **OS1** and **OS2**, shown in Figure 11, were established by NMR spectroscopy, as described above for the O-polysaccharide, (for the ^1^H- and ^13^C-NMR chemical shifts, see Table 3), and electrospray ionisation MS. The negative ion HR ESI mass spectrum showed a [M-H]^−^ peak of C_21_H_34_O_19_ at m/z 589.1651 (calculated value 589.1694) and a [M-H]^−^ peak of C_24_H_40_O_21_ at m/z 663.2034 (calculated value 663.2062).

**Figure 12:**
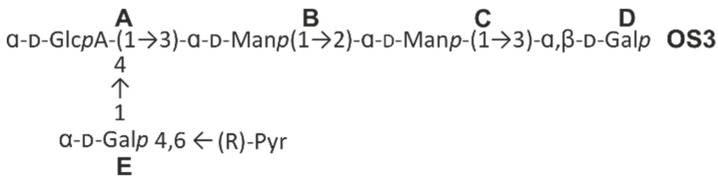
Structure of the oligosaccharide (**OS3**) after bacteriophage RBP2 treatment.

**Figure 13:**
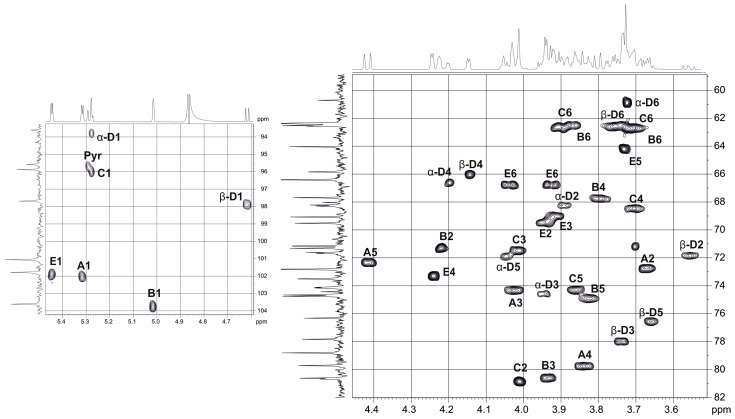
Parts of the ^1^H,^13^C HSQC spectrum of the **OS3**. Arabic numerals refer to carbons of sugar residues as designated in Table 3.

The products of the cleavage of the *Kpn* K21 capsular polysaccharide with depolymerase RBP2 were fractionated by gel permeation chromatography to give oligosaccharide (**OS3**) (Figure 12). The structure of the oligosaccharide was established by 1D and 2D NMR spectroscopy. The ^13^C NMR spectrum of **OS3** (see Figure 13) contained signals of five anomeric atoms of linked monosaccharides (units **A**, **B**, **C** and **E**), at δ 102.1, 103.7, 95.9 and 101.9 and one monosaccharide at the reducing end (unit **D**) at δ 93.8 and 103.8 (for α- and β-anomer, **Dα** and **Dβ**, respectively). Oligosaccharide **3** was shown to be a pentasaccharide (see Figure 13 and Table 3). These data indicated that the phage depolymerase was glycosidase that specifically cleaved the β-Gal-(1→3)-α–GlcA linkage between K21 units in the CPS.

## 3. Discussion

The capsular polysaccharide of *Klebsiella* is an important virulence factor of the bacterium. Finding phages capable of depolymerising this polysaccharide is, thus, an important task in the development of methods to manage *Kpn* infections. The production of recombinant RBPs both enables an expansion of the understanding of the process of phage recognition of cells and provides a powerful tool in the fight against *Kpn* and its biofilms.

This study has described *Klebsiella* phage K5, which is active against *Klebsiella* strains with the K21 capsular polysaccharide type. The Pathogen watch database (https://pathogen.watch/, accessed on 1 November 2023) contains 246 genomes with this capsular type isolated in various geographical areas around the world, which indicates the clinical significance of strains with this capsular type. The isolated phage has the ability to depolymerise the host CPS and causes rapid lysis of the *Kpn* 5 culture.

The genome of phage K5 encodes two enzymes with predicted polysaccharide-degrading activity. Previous studies described a closely related bacteriophage KP32, which has two tail spikes that degrade CPS and form a branching structure [28]. In addition, each of the two tail spike RBPs is active against its own type of capsular polysaccharide, namely K3 and K21 for KP32gp37 and KP32gp38, respectively [27]. It can be proposed that the virion of *Klebsiella* phage K5 also has two tail spikes, RBP1 and RBP2.

BLASTp analysis revealed that the depolymerase RBP2 was almost identical to KP32gp38. Experimental data confirmed that recombinant RBP2 also depolymerises the K21 type of capsular polysaccharide.

At the same time, RBP1 is homologous to the tail spikes of phages KMI2, KMI1 and KMI4 not studied comprehensively. Unlike KP32gp37, recombinant RBP1 was inactive against CPS type K3 and showed no activity with any of the types of capsular polysaccharide tested. The host range of *Klebsiella* phage K5, which only infects the isolation host with CPS type 21, is consistent with these data. Thus, the CPS against which this enzyme may be active remains unknown.

This study has demonstrated the structure of *Klebsiella* strain 5 representing type K21 of capsular polysaccharide. The structure of this capsule type was previously studied back in the early 70s, by Choy and Dutton [36]. The structure obtained confirms, and is consistent with, data published in the literature.

The goal of the study was to determine that the linkages in the polysaccharide are degraded by RBP2 of phage K5, and the type of CPS fragments that are then produced. The type K21 CPS from *Kpn* was treated with RBP2 and only one carbohydrate fraction was obtained after purification. The oligosaccharide obtained was studied using ^13^C and ^1^H NMR spectroscopy and shown to be a pentasaccharide corresponding to a monomer of the repeated unit. It was found that the cleavage occurred at the β-galactosidic bond between β-d-Gal*p* and α-d-Glc*p*A.

**Figure 14:**
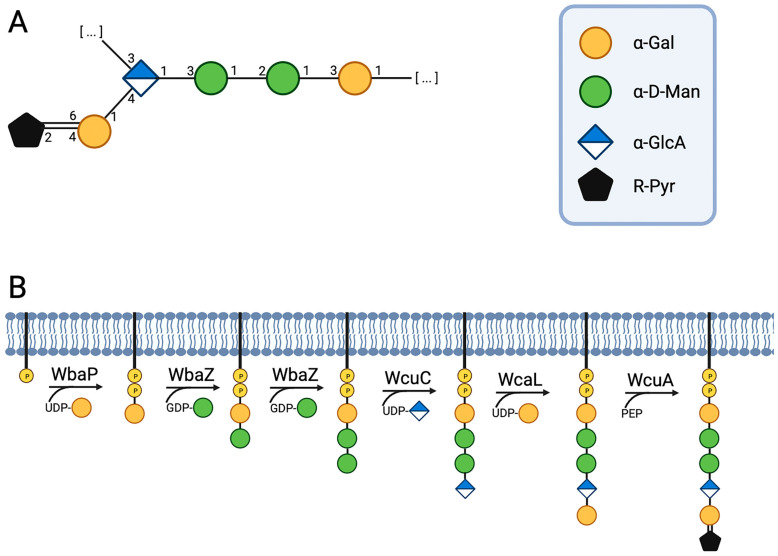
Putative path of the CPS K21 biosynthesis. (**A**) Schematic representation of the structure of the polysaccharide. (**B**) Scheme of the proposed polysaccharide biosynthesis pathway. The image was created using the service https://www.biorender.com/.

Data on the gene composition of CPS synthesis clusters of *K. pneumoniae* and their NMR structure have been compiled and reported previously [8]. Based on gene occurrence, correlations between specific glycosyl transferase genes and chemical bonds were determined and the most probable transferase chemical activities were identified [8]. To the authors’ knowledge, however, the biosynthesis of capsular polysaccharide K21 has not yet been described in detail. An attempt was made to fill this gap (Figure 14). Based on previous identification, it can be supposed that the first step in polysaccharide biosynthesis is the transfer of b-d-Galp to the undecaprenyl phosphate molecule by the initiating transferase WbaP. In the second step, WbaZ transfers the a-d-Manp residue onto a disaccharide, creating the α-d-Manp-(1-3)-β-d-Galp linkage. Transferase WcuC then adds the α-d-GlcpA residue, forming the α-d-GlcpA-(1-3)-α-d-Manp linkage. It was found that the transferase gene WcaL is unique and is present only in the KL21 synthesis gene cluster [8]; the study demonstrated that it is also present in the KL154 cluster [9]. Accordingly, the authors believe that this transferase organises the final a-d-Galp-(1-4)-α-d-GlcpA linkage by transferring the α-d-Galp residue onto a terasaccharide. Finally, a pyruvyltransferase WcuA adds an R-pyruvate residue to the α-d-Galp pentasaccharide at positions 4 and 6.

## 4. Materials and Methods

Overall design of the experiment is presented in Supplementary Figure S1.

### 4.1. Klebsiella Phage K5: Isolation and Growth Conditions

The *K. pneumoniae* strain 5 (JAWJEB000000000) with capsule type K21 was used to isolate *Klebsiella* phage K5 from wastewater collected in the Moscow region (Russia). A standard enrichment culture protocol was used for isolation [37]. An enrichment mixture was titrated using the double layer technique on LB nutrient medium (tryptone—10 g/L, yeast extract—5 g/L, NaCl—10 g/L) using 1.5% agar for a bottom layer and 0.75% agar for a top layer [38].

The phage obtained from a single PFU was propagated using the isolation host in a volume of 400 mL. The resulting lysate was treated with chloroform and centrifuged (8000× *g*, 20 min) to remove cellular debris. The resulting supernatant was then concentrated with PEG 8000, treated with DNase A, and pelleted at 16,500× *g* for 2 h. The resulting pellet was resuspended in 1 mL of SM buffer (NaCl—100 mM, MgSO_4_x7H_2_O—8 mM, Tris-HCl—50 mM, pH = 7.0) and stored at +4 °C.

### 4.2. One-Step Growth and Adsorption Curves

To determine adsorption time, the host cells were grown at 37 °C until OD_600_ = 0.3. Then, a suspension of phage with a multiplicity of infection (MOI) of 0.001 was added to the cells.

Samples in 100 µL were taken after 1, 2, 3, 4, 5, 8, 10, 15 and 20 min of incubation and added to 900 µL of ice-cold buffer, with the addition of 50 µL of chloroform. The selected samples were immediately centrifuged at 8000× *g* and immediately titrated.

In the one-step growth experiment, 50 mL of host cells (OD600 = 0.3) were pelleted by centrifugation (8000× *g*, 10 min, 4 °C) and resuspended in 0.5 mL of heated LB medium. Next, bacteriophage with an MOI of 0.01 was added to the resuspended cells and incubated for 5 min at 37 °C to adsorb phage particles on the cells. Unbound phage was removed by centrifugation, and the cells were resuspended in a heated medium to the original volume. Thereafter, samples were taken every 5 min for two hours and analysed as described above.

Both experiments were carried out in three biological replicates and each sample was analysed twice. To plot the curve, the mean value was used, with the standard deviation indicated.

### 4.3. Host Range

A total of 35 clinical isolates of *K. pneumoniae* with various capsular types were tested to determine the host range of *Klebsiella* phage K5. The list of strains is shown in Table 1.

The spot test was used for the primary testing of the lytic activity of the bacteriophage. On a Petri dish with a double agar, containing cells of the tested strain in the upper layer, 10 µL of a phage suspension with a concentration of 10^8^ PFU/mL was applied and incubated overnight at 37 °C. The presence of lysis was determined by the appearance of a transparent spot in the area of application of the phage suspension. If a transparent spot appeared, the bacteriophage was additionally titrated using the test strain until single plaques appeared.

### 4.4. Phage Genome Sequencing and Annotation

The genome of strain 1053 was sequenced in 2015, using 454 pyrosequencing technology with 19-fold coverage and a median length of 910 bp, with a Roche 454 Life Science Genome Sequencer FLX+ (Roche, Basel, Switzerland). A library was constructed using Rapid Library Preparation Kit and Rapid Library MID Adaptors Kit. Emulsion PCR and sequencing were performed using GS FLX Titanium LV emPCR Kit (Lib-L), GS FLX Titanium emPCR Breaking Kits LV/MV, GS FLX Titanium Sequencing Kit XL+ and GS FLX Titanium PicoTiterPlate Kit 70 × 75. All procedures were carried out according to the protocols of the manufacturer.

De novo genome assembly was performed using CLC Genomic Workbench 23 (QIAGEN, Aarhus, Denmark). A search for open-reading frames (ORF) was conducted using v1.13.4 [39], Glimmer v3.0.2 [40] and Prodigal v2.6.3 [41]. ORF boundaries were curated manually. Gene functions were predicted with BLAST v2.13.0 [42] and HHpred server [43] taking into account the recommendations set out in [44]. The BLAST search employed the NCBI nr/nt databases and the HHpred search used PDB70_mmcif_2023-06-18, PfamA-v35, UniProt-SwissProt-viral70_3_Nov_2021 and NCBI_Conserved_Domains(CD)_v3.19 databases. The presence of tRNA genes was checked using tRNAscan-SE v2.0 [45] and ARAGORN v1.2.38 [46]. The annotated genome of *Klebsiella* phage K5 has been deposited in the NCBI GenBank under accession number KR149291.

### 4.5. Genome Analysis and AlphaFold Modelling

Intergenomic comparisons and calculations of intergenomic similarities were performed using clinker v0.0.28 [47] and VIRIDIC v1.1 [25] with default settings. Genetic maps and gene comparisons were visualised in clinker. Protein sequences alignments were carried out using Clustal Omega v1.2.4 [48] and “number of refinement iterations 3, evaluate full distance matrix for initial guide tree, evaluate full distance matrix for refinement iteration guide tree” command line parameters. Phylogenetic analysis was performed using IQ-TREE v2.2.5 [49] and “–alrt 1000-B 5000” command line parameters. The resulting consensus trees with bootstrap support values (1000 replicas) were visualised using iTOL v6 [50]. Protein structures were modelled with AlphaFold 2.2.4 using full databases and the command line parameter or “–multimer”.

### 4.6. Tail Spike Protein Cloning, Expression and Purification

Genes encoding both tail spikes of *Klebsiella* phage K5 RBP1 (gp40) and RBP2 (gp41) were PCR-amplified using primers 5.1.1.f:ATAGGATCCAACGACCCGGCGTCTCTT/5.1.1.R:TATAAGCTTAATAGTTATAAGCAACAAATCG for RBP-1 and 5.1.2.f:ATAGGATCC CCACGTTTCAATCAGCCGA/5.1.2.R:TATAAGCTTATGAGACGAATGCTCTTAC for RBP-2 with generated BamHI and HindIII cloning sites. The primers were synthesised by Evrogen (Moscow, Russia). The amplified genes were cloned to pTSL vector (GenBank: KU314761.1). The accuracy of the insert was verified by PCR, using the same primers and Sanger sequencing of the plasmid region flanked by T7 primers.

Protein expression was carried out in *E. coli* B834(DE3). After induction with 1 mM IPTG, the culture was incubated overnight at 18 °C. Cells were centrifuged at 4000× *g*, resuspended in a 20 mM Tris-HCl (pH 8.0), 200 mM NaCl buffer, disrupted by ultrasonic treatment (Virsonic, VirTis, Stone Ridge, NY, USA), and then the cell debris was removed by centrifugation at 13,000× *g*.

The protein products were purified on a Ni-NTA Sepharose column (5 mL, GE Healthcare, Chicago, IL, USA) by 50–400 mM imidazole step gradient in 20 mM Tris-HCl (pH 8.0), 200 mM NaCl.

The purified protein was collected and dialysed with 20 mM Tris-HCl buffer (pH 8.0). TEV protease was used for a 12 h incubation at 20 °C to cleave SlyD and His-tag.

The target protein was finally purified on a 5 mL SourceQ 15 (GE Healthcare, Chicago, IL, USA), using a linear gradient of 0–600 mM NaCl in 20 mM Tris-HCl (pH 8.0). Protein concentration was determined spectrophotometrically at 280 nm, using a calculated molar extinction coefficient of 125,250 M^−1^ cm^−1^.

### 4.7. Spot Assay

Bacterial lawns were prepared using a double layer technique, as described in Section 4.1. After applying 10 μL of the purified protein to the surface of the bacterial lawn, it was allowed to dry and was incubated overnight at 37 °C. A cleared spot on the site of application was used to determine the presence of activity.

### 4.8. Isolation of Capsular Polysaccharides

Capsular polysaccharides were isolated from bacterial cells of *K. pneumoniae* 5 by the phenol-water method [31]. The crude extract was dialysed without separation of the layers and freed from nucleic acids and proteins by treatment with 50% aq CCl_3_CO_2_H to pH 2 at 4 °C. The supernatant was dialysed and lyophilised. To remove lipooligosaccharide-derived minor impurities, a CPS sample (294.9 mg) was hydrolysed with 2% CH_3_CO_2_H (100 °C, 2 h), and the products were fractionated by gel-permeation chromatography on a column (56 × 2.5 cm) of Sephadex G-50 Superfine (GE Healthcare, Chicago, IL, USA) in 0.05 M pyridinium acetate pH 4.5 as eluent to give a purified CPS sample (53 mg). The yield of the capsular polysaccharide was 18% of dried cells mass.

### 4.9. Isolation of the O-Polysaccharide

Depyruvation of polysaccharide from *K. pneumoniae* strain 5 (60 mg) was performed with 2% aq HOAc at 100 °C for 7 h. The precipitate was removed by centrifugation (13,000× *g*, 20 min) and the supernatant was fractionated by GPC on a column (80 × 1.6 cm) of TSK HW-40 (S) in 1% HOAc in 1% acetic acid, monitored with a differential refractometer (Knauer, Berlin, Germany). The O-polysaccharide without pyruvate group was obtained in yields of 38% (23 mg) of the polysaccharide mass.

### 4.10. Smith Degradation

The polysaccharide (19.5 mg) was oxidised with sodium metaperiodate (21.7 mg in 1 mL of water), in the dark, for 72 h at 20 °C; then, the product was reduced by an excess of NaBH_4_ (53 mg) and desalted on a column (80 × 1.6 cm) of TSK HW-40 (S) (Toyo Soda, Tokyo, Japan) in 1% HOAc in 1% acetic acid. The resultant modified polysaccharide was hydrolysed with 2% acetic acid for 2 h at 100 °C, and the products were fractionated on a (80 × 1.6 cm) of TSK HW-40 gel column in 1% acetic acid to give a mixture of two oligosaccharides (18.4 mg).

### 4.11. Monosaccharide Analysis

A sample of the O-polysaccharide from strain 05 (1 mg) was hydrolysed with 2 M CF_3_CO_2_H (120 °C, 2 h). Neutral monosaccharides were identified by GLC of the alditol acetates on a Maestro 7820 GC instrument (Interlab, Moscow, Russia) equipped with a HP-5ms column, using a temperature programme of 160 (1 min) to 290 °C at 7 °C min^−1^. The absolute configurations of the monosaccharides were determined by GLC of the acetylated (*S*)-2-octyl glycosides as described.

### 4.12. NMR Spectroscopy

Samples were deuterium-exchanged by freeze-drying from 99.9% D_2_O and then examined as solutions in 99.95% D_2_O. NMR spectra were recorded on a Bruker Avance II 600 spectrometer (Bruker Daltonics, Bremen, Germany) at 55 °C, using internal sodium 3-(trimethylsylil)propanoate-2,2,3,3-d_4_ (δ_H_ 0, δ_C_ -1.6) as a reference for calibration. The 2D NMR spectra were obtained using standard Bruker software as the Bruker TopSpin 3.6.0 program, and the Bruker TopSpin 2.1 program was used to acquire and process the NMR data. A mixing time of 100 and 150 ms was used in TOCSY and ROESY experiments, respectively.

## Figures and Tables

**Table 1:** Host range of *Klebsiella* phage K5.

N°	Strain	K Type	Lysis
1	*K.pneumoniae* 62867	K1	-
2	*K.pneumoniae* 77245	K2	-
3	*K.pneumoniae* 69402	K12	-
4	*K.pneumoniae* KL12	K12	-
5	*K.pneumoniae* KL13	K13	-
6	*K.pneumoniae* kot L	K14	-
7	*K.pneumoniae* 88166	K15	-
8	*K.pneumoniae* KphM	K16	-
9	*K.pneumoniae* 77840	K16	-
10	*K.pneumoniae* 74610	K17	-
11	*K.pneumoniae* 80384	K20	-
12	*K.pneumoniae* Kph1	K20	-
13	*K.pneumoniae* K5 (isolation host)	K21	+
14	*K.pneumoniae* Kph5	K23	-
15	*K.pneumoniae* 78315	K24	-
16	*K.pneumoniae* 77680	K25	-
17	*K.pneumoniae* 77487	K39	-
18	*K.oxytoca* 1620	K55	-
19	*K.pneumoniae* 81841	K62	-
20	*K.pneumoniae* 77864	K64	-
21	*K.pneumoniae* Kph13	K107	-
22	*K.pneumoniae* 1333	K108	-
23	*K.pneumoniae* 1226	K112	-
24	*K.pneumoniae* 19.01	K114	-
25	*K.pneumoniae* 15	K161	-
26	*K.pneumoniae* 3	Uncharacterised	-
27	*K.pneumoniae* 1	Uncharacterised	-
28	*K.pneumoniae* 3.1	Uncharacterised	-
29	*K.pneumoniae* OX2	Uncharacterised	-
30	*K.pneumoniae* 1481	Uncharacterised	-
31	*K.pneumoniae* 224	Uncharacterised	-
32	*K.pneumoniae* OX140	Uncharacterised	-
33	*K.pneumoniae* 203	Uncharacterised	-
34	*K.pneumoniae* 186	Uncharacterised	-
35	*K.pneumoniae* 197	Uncharacterised	-

**Table 2:** Chemical shifts in the ^1^H and ^13^C NMR spectra (δ, ppm). Structures of the *Kpn* K21 CPS and MPS are shown in Figure 9. ^1^H NMR chemical shifts are shown in italics.

Monosaccharide Residue		C1	C2	C3	C4	C5	C6
		*H1*	*H2*	*H3*	*H4*	*H5*	*H6* (*6a*, *6b*)
**CPS**							
**→**3,4-α-d-Glc*p*A-(1**→**3	**A**	101.5	73.2	80.5	71.3	74.1	175.1
		*5.25*	*3.87*	*4.31*	*3.92*	*4.21*	–
**→**3-α-d-Man*p*-(1**→**2	**B**	103.3	70.9	79.6	74.5	74.6	62.3
		*5.02*	*4.19*	*3.91*	*3.79*	*3.71*	*3.85*; *3.74*
**→**2-α-d-Man*p*-(1**→**3	**C**	95.9	80.3	71.4	68.3	76.8	62.3
		*5.23*	*4.01*	*3.97*	*3.68*	*3.90*	*3.85*; *3.74*
**→**3-β-d-Gal*p*-(1**→**3	**D**	104.2	71.2	77.8	65.9	76.4	62.3
		*4.85*	*3.66*	*3.76*	*4.13*	*3.64*	*3.87*
(4,6)-Pyr-α-d-Gal*p*-(1**→**4	**E**	100.7	69.5	69.2	72.6	63.9	66.1
		*5.50*	*3.88*	*3.84*	*4.17*	*3.72*	*3.98*, *3.85*
4,6-pyruvate			*5.33*	*1.44*			
		176.3	101.6	26.4			
**MPS**							
**→**3,4-α-d-Glc*p*A-(1**→**3	**A**	101.7	73.2	80.5	75.7	74.1	175.1
		*5.23*	*3.88*	*4.37*	*3.93*	*4.28*	-
**→**3-α-d-Man*p*-(1**→**2	**B**	103.4	71.0	79.7	67.5	74.6	62.3
		*5.03*	*4.23*	*3.93*	*3.82*	*3.81*	*3.85*; *3.74*
**→**2-α-d-Man*p*-(1**→**3	**C**	95.8	80.5	71.3	68.3	74.1	62.3
		*5.23*	*4.01*	*3.99*	*3.67*	*3.88*	*3.85*; *3.74*
**→**3-β-d-Gal*p*-(1**→**3	**D**	104.2	71.2	77.7	65.8	76.4	62.3
		*4.89*	*3.65*	*3.78*	*4.15*	*3.65*	*3.87*
α-d-Gal*p*-(1**→**4	**E**	99.7	69.8	70.6	70.2	72.0	62.3
		*5.58*	*3.80*	*3.80*	*3.98*	*3.90*	*3.87*

**Table 3:** Chemical shifts in the ^1^H and ^13^C NMR spectra (δ, ppm). Oligosaccharides were derived from the *Kpn* K21 CPS and MPS by Smith degradation (products **OS1** and **OS2**) and by bacteriophage treatment (**OS3**). ^1^H NMR chemical shifts are shown in italics.

Monosaccharide Residue	C1		C2	C3	C4	C5	C6
	*H1*		*H2*	*H3*	*H4*	*H5*	*H6* (*6a*, *6b*)
**OS1**							
**→**3-α-d-Glc*p*A-(1**→**3	**A**	101.9	72.4	81.9	71.3	73.0	175.9
		*5.28*	*3.82*	*4.06*	*4.07*	*4.29*	*-*
**→**3-α-d-Man*p*-(1**→**2	**B**	100.3	71.1	80.5	67.0	74.2	62.5
		*5.07*	*4.16*	*3.94*	*3.87*	*3.92*	*3.93*; *3.90*
**→**3-β-d-Gal*p*-(1**→**3	**D**	104.4	72.5	73.8	69.9	76.5	62.4
		*4.67*	*3.59*	*3.67*	*3.91*	*3.71*	*3.86*
Gro	**C**	90.3	81.1	62.3			
		*5.13*	*3.67*	*3.75*			
**OS2**							
**→**3-α-d-Glc*p*A-(1**→**3	**A**	101.9	72.4	81.9	71.3	73.0	175.9
		*5.28*	*3.82*	*4.06*	*4.07*	*4.29*	*-*
**→**3-α-d-Man*p*-(1**→**2	**B**	100.5	71.0	80.6	67.0	74.2	62.5
		*5.04*	*4.17*	*3.98*	*3.87*	*3.92*	*3.93*; *3.90*
**→**3-β-d-Gal*p*-(1**→**3	**D**	104.4	72.5	73.8	69.9	76.5	62.4
		*4.67*	*3.59*	*3.67*	*3.91*	*3.71*	*3.86*
Aglycone	**C**	103.9	77.6	62.8	63.0	77.9	67.6
		*5.13*	*3.90*	*3.76*	*3.72*; *3.61*	*4.29*	*4.02*; *3.81*
**OS3**							
**→**3,4-α-d-Glc*p*A-(1**→**3	**A**	102.1	72.8	74.4	79.7	72.4	174.8
		*5.32*	*3.67*	*4.03*	*3.84*	*4.42*	
**→**3-α-d-Man*p*-(1**→**2	**B**	95.9	71.3	80.6	67.8	74.9	62.5
		*5.28*	*4.22*	*3.93*	*3.79*	*3.83*	*3.89*; *3.72*
**→**2-α-d-Man*p*-(1**→**3	**C**	96.0	80.7	71.4	68.5	74.3	62.5
		*5.23*	*4.01*	*4.01*	*3.70*	*3.86*	*3.88*; *3.77*
**→**3-β-d-Gal*p*-(1**→**3	**D**β	103.8	71.9	78.0	66.0	76.5	62.4
		*5.02*	*3.56*	*3.74*	*4.14*	*3.66*	*3.76*
**→**3-α-d-Gal*p*-(1**→**3	**D**α	93.8	68.3	74.5	66.6	71.9	61.0
		*5.28*	*3.89*	*3.95*	*4.20*	*4.05*	*3.72*
(4,6)-Pyr-α-d-Gal*p*-(1**→**4	**E**	101.9	69.5	69.0	73.3	64.3	66.8
		*5.45*	*3.94*	*3.91*	*4.24*	*3.73*	*4.04*; *3.93*
4,6-pyruvate		176.7	95.6	26.4			
			*5.29*	*1.55*			

## Data Availability

The annotated genomic sequence of Klebsiella phage K5 has been deposited to GenBank and is available under accessions #KR149291 for initial submission in 2015 and #NC_028800 for refined re-annotated version in 2023. The genome of the bacterial host Klebsiella 5 has been deposited to NCBI GenBank and is available under accession number JAWJEB000000000.

## Supplementary Materials

The supporting information can be downloaded at https://www.mdpi.com/article/10.3390/ijms242417288/s1.
