# Supplementary figures and images for: Depolymerisation of the Klebsiella pneumoniae Capsular Polysaccharide K21 by Klebsiella Phage K5
Source: Int J Mol Sci. 2023 Dec 9;24(24):17288. doi: 10.3390/ijms242417288 (PMC10743669; doi:10.3390/ijms242417288)

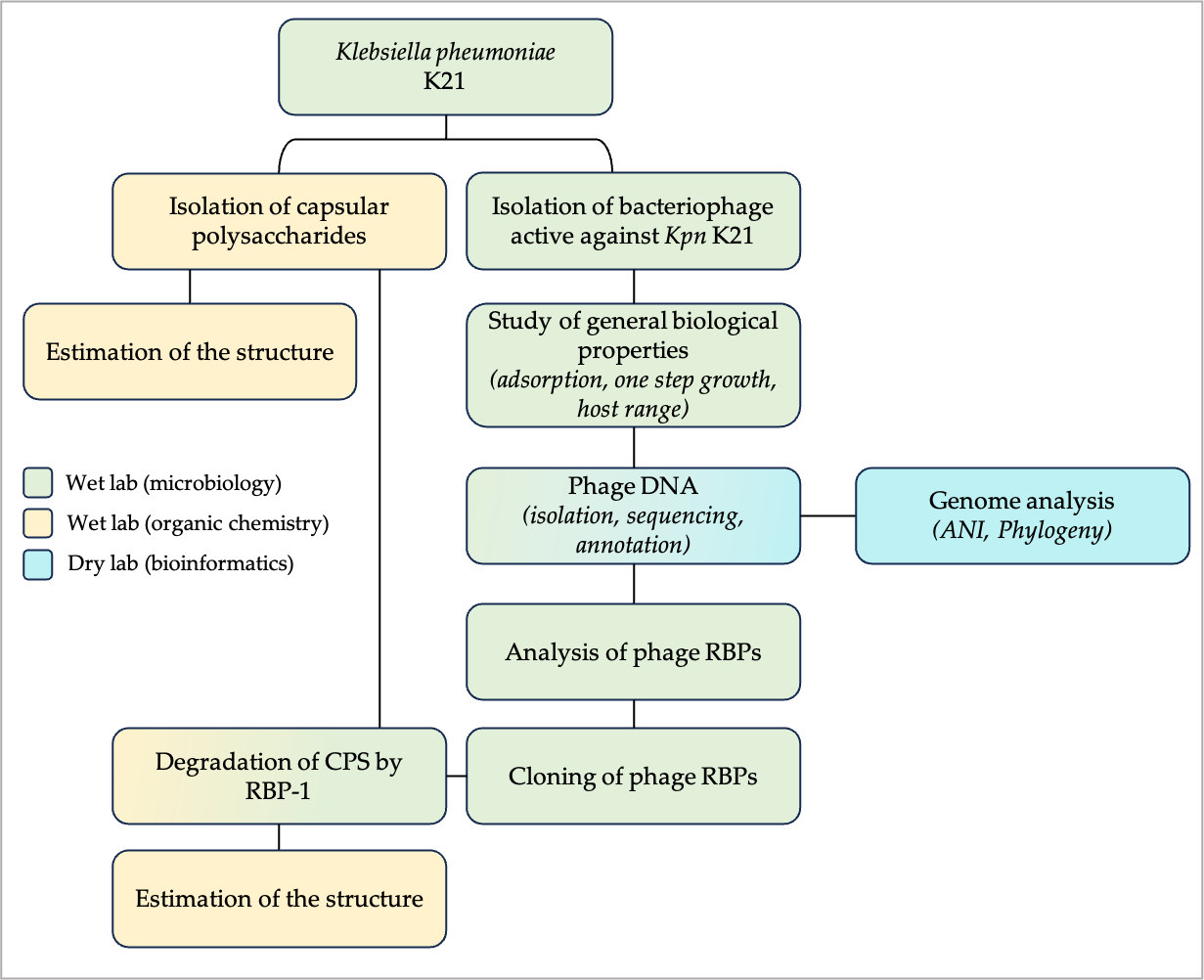

Supplement: Supplementary file 1 [file ijms-24-17288-s001.zip › Suppl figure S1.png]
